# Prognostic Factors Influencing Survival in Stage II and Stage III Colorectal Cancer Patients

**DOI:** 10.7759/cureus.46575

**Published:** 2023-10-06

**Authors:** Mohammad S Alkader, Murad Z Al-Majthoub, Walid A Al-Qerem, Doa'a M Alkhader, Aseel M Alhusban, Muna A Abdulkareem, Bashar Abweny, Alaa T Hamawi, Hala F Muslem, Rasha A Omeish, AlAnoud M Al-Adwan, Hamed A Al Halaiqah

**Affiliations:** 1 Department of Medical Oncology, Jordanian Royal Medical Services, Amman, JOR; 2 Department of Internal Medicine, Jordanian Royal Medical Services, Amman, JOR; 3 Department of Pharmacy, Al-Zaytoonah University of Jordan, Amman, JOR; 4 Department of Gastroenterology and Hepatology, Jordanian Royal Medical Services, Amman, JOR; 5 Department of Dermatology, Jordanian Royal Medical Services, Amman, JOR; 6 Department of Internal Medicine, Mutah University, Alkarak, JOR

**Keywords:** jordan, prognosis, chemotherapy, disease-free survival (dfs), overall survival (os), colorectal cancer

## Abstract

Background

Colorectal cancer (CRC) is a global health concern with rising incidence. This study analyzed demographic and clinicopathological factors influencing overall survival (OS) and disease-free survival (DFS) in Jordanian CRC patients.

Methodology

This retrospective, single-center study collected data from CRC patients at the Royal Medical Services, Jordan, from January 2018 to June 2020. Patient variables included disease stage, stage risk, tumor location, history of chemotherapy, and metastasis status. OS and DFS were defined as the time from surgery to death, last follow-up, or metastasis confirmation. Kaplan-Meier curves and Cox models were used for survival analysis.

Results

Of 127 CRC patients, 33.3% died during the follow-up period. Most patients were males (55.1%), diagnosed with stage III (55.9%), and classified as high risk (59.2%). Metastasis occurred in 24.4%, and 65.4% received chemotherapy. OS at one, two, and end of the follow-up years was 85.2%, 75.6%, and 66.9%, respectively. Metastasis-free rates were 85%, 78.5%, and 71%, respectively. Multivariate analysis showed that stage III (hazard ratio (HR) = 2.968) and high-risk stage (HR = 2.966) were associated with shorter OS and increased metastasis risk. Right-sided tumors (HR = 2.183) had shorter OS, while chemotherapy recipients (HR = 0.430) had longer OS. Stage III and high-risk stages were strong predictors of mortality, while only stage III and high-risk stages were robust predictors of metastasis. Demographic variables (sex and age) showed no significant associations with survival outcomes.

Conclusions

Our findings highlight the prognostic significance of disease stage, stage risk, tumor location, and chemotherapy in CRC survival among Jordanian patients. Understanding these factors can guide tailored treatment and improve outcomes.

## Introduction

Colorectal adenocarcinoma, or colorectal cancer (CRC), is the third leading cause of cancer-related deaths worldwide and the second leading cause of cancer-related death in the United States, with rapidly increasing occurrence in developed countries [1]. CRC usually originates from glandular epithelial tissue of the large intestines. CRC is highly attributed to genetic and epigenetic mutations that provide cancerous tissue progressive proliferation and prolonged survival [2].

CRC comprises 11% of cancer diagnoses according to GLOBOCAN 2020 data, with higher incidence in men, and three to four times higher incidence in developing countries [3]. Of the Middle Eastern countries, CRC is the deadliest cancer in males in Saudi Arabia, the United Arab Emirates (UAE), and Oman [4]. In Jordan, CRC ranks as the number one cancer among men and the second most common cancer among women, accounting for 15% and 9.4%, respectively, of all male and female diagnosed cancers [5].

Advancements in CRC treatment have resulted in reduced mortality rates in developing countries despite the increased incidence rates [4]. Although the introduction of more effective screening tests may have contributed to higher incidence rates by identifying previously undiagnosed cases, in the long term, these tests have led to decreased death rates by identifying and removing pre-cancerous or non-metastasized cells [6]. Unfortunately, Jordan lacks a national screening program for detecting the disease in high-risk patients using a colonoscopy or a fecal occult blood test.

Risk factors of CRC range from non-modifiable risks such as race and ethnicity, with higher risk and lower survival rates in African and Native Americans, in addition to 1.5 higher risk in males. Hereditary non-polyposis colorectal cancer and familial adenomatous polyposis account for 7-10% of CRC cases with an autosomal dominant pattern of inheritance [7]. In contrast, the modifiable risk factors include physical inactivity, obesity, diet, smoking history, and diabetes mellitus [8].

According to the most recent extensive report on cancer occurrence in 2012, CRC constituted 11.3% of newly identified cases within the Jordanian population, positioning it as the second most prevalent cancer across both males and females [9]. The general survival rates at the five-year and decade marks for colorectal cancer stood at 58.2% and 51.8%, respectively. Reduced survival rates were linked to older age, limited cell differentiation, progressed cancer stages, and cancers on the right side. Implementing screening tactics becomes crucial for the timely identification of colon adenomas and colorectal cancer in the Jordanian context [9].

Previous studies have demonstrated the risk factors associated with CRC survival in Jordan; however, to our knowledge, no study has investigated the effects of chemotherapy on CRC survival.

In this retrospective, single-center, observational study, we aimed to describe a population of CRC patients in Jordan and investigate demographic and clinicopathological predictors of overall survival (OS) and disease-free survival (DFS).

## Materials and methods

Study design

We conducted a retrospective, single-center, observational study at the Royal Medical Services in Jordan. Data from patients with CRC were retrospectively collected through hospital records between January 2018 and June 2020. Patients were excluded if they had incomplete medical records, lost to follow-up, showed the presence of another primary tumor, were in stage I or IV, had other comorbidities such as end-stage kidney disease or liver cirrhosis, showed poor Eastern Cooperative Oncology Group (ECOG) score, deceased within 30 days postoperatively, or if they received adjuvant chemotherapy with less than two months duration.

All patients included in the analysis were treated with adjuvant chemotherapy, and had received one of the following regimens: (1) mFOLFOX6 regimen (oxaliplatin 85 mg/m^2^ first day, folinic acid 400 mg/m^2^ first day, 5-fluorouracil (5-FU) 400 mg/m^2^ intravenous bolus first day, 5-FU 2,400 mg/m^2^ 46 hours continuous infusion) was applied every two weeks; (2) single-agent capecitabine (850-1,000 mg/m^2^ for 14 days every three weeks); (3) combination of 5-FU (500 mg/m^2^ intravenous bolus weekly for six weeks every eight weeks) and folinic acid (500 mg/m^2^ intravenous bolus weekly for six weeks every eight weeks); and (4) XELOX regimen (oxaliplatin 130 mg/m^2^ first day, capecitabine 850 mg/m^2^ for 14 days, repeated every three weeks).

Patient consent was waived from the institutional review board due to the retrospective nature of the study. The study was conducted in accordance with the ethical guidelines of Helsinki.

Data collection

Demographic data were collected for age and sex. Clinicopathological characteristics included the American Joint Cancer Committee disease stage, stage risk (low or high), tumor location, history of chemotherapy, and metastasis status. Following the guidelines provided by the National Comprehensive Cancer Network, stage II tumors classified as high-risk encompass attributes such as poorly differentiated/undifferentiated histology, lymphatic/vascular invasion, bowel obstruction, examination of fewer than 12 lymph nodes, perineural invasion, localized perforation, close, indeterminate, or positive margins, or a high degree of tumor budding [10]. For patients in the high-risk category of stage II, there is a lack of available data that establishes a correlation between risk factors and the choice of chemotherapy. While stage III tumors, the low-risk stage was defined as tumors with T1-3, N1 while the high-risk stage was defined as tumors with T4, N1-2, or any T with N2.

Study outcomes

Primary outcomes of our study included OS defined as the time from surgery until the time of death or last follow-up or end of the study period. DFS was defined as the time from surgery until the date of radiological or histopathological confirmation of metastasis.

Statistical analysis

Categorical variables were described using frequencies (percentages, %) and continuous variables were described as median (95% Cl). Survival curves were produced using the Kaplan-Meier method for each predictor for OS and DFS. Univariate Cox models were employed to fit predictors for each of the survival outcomes. The proportional hazards (PH) assumption test was carried out by applying the cox.zph function in R (version 4.2.2) with the default “km” function. An assessment of the PH assumption was initially conducted by examining all baseline variables that had a p-value <0.2 in the univariate Cox models except for the varying variable (receiving chemotherapy) and were included as independent variables in the multivariate analysis. A Cox model with a time-varying effect assuming piece-wise constant hazard ratios (HRs) was employed to assess variables associated with OS. A multivariate Cox model was built to assess variables associated with disease-free survival. A p-value <0.05 was considered significant.

## Results

Table 1 presents the baseline of demographic and clinicopathological characteristics of included patients. Among the cohort of 127 CRC patients, 42 (33.3%) individuals died by the end of the follow-up period. The median age of the enrolled patients was 62 years (59-65 years), with a predominance of males (55.1%). Furthermore, 55.9% of the patients were diagnosed with stage III, and a significant proportion (59.2%) were classified as high risk. Additionally, 31 (24.4%) patients experienced at least one occurrence of metastasis, and 83 (65.4%) patients had received adjuvant chemotherapy.

**Table 1:** Baseline characteristics of included patients.

Variable	Median (95%CI) or Frequency (%)
Age	62 (59–65)
Gender	
Male	70 (55.1%)
Female	57 (44.9%)
Stage	
II	56 (44.1%)
III	71 (55.9%)
Risk of the stage	
Low risk	42 (40.8%)
High risk	61 (59.2%)
Site	
Left	78 (61.4%)
Right	49 (38.6%)
Death	42 (33.1%)
Overall survival for deceased participants	469 (329-757)
Chemotherapy	83 (65.4%)
Metastasis	31 (24.4%)
Disease-free survival	356 (200-489)

**Figure 1:**
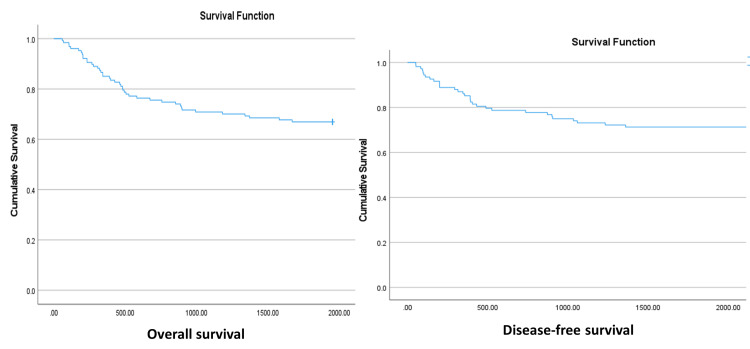
Survival patterns over time.

Kaplan-Meier curves for the outcome measures examined are shown in Figure 1. The OS was 85.2% by the end of the first year after the surgery, 75.6% by the end of the second year, and 66.9% by the end of the follow-up duration (OS: Figure 1a). Figure 1b shows that 85% of the patients were metastasis-free by the end of the first year, 78.5% by the end of the second year, and 71% by the end of the follow-up period.

The multivariate models are presented in Table 2 and Table 3. The stages of cancer and their associated risk levels demonstrated significant associations with OS and DFS. Specifically, being in stage III was associated with shorter OS time (HR = 2.968, 95% CI = 1.460-6.036, p = 0.003) and with an increased recurrence of metastasis (HR = 3.837, 95% CI = 1.538-9.575, p = 0.004). Patients with high-risk criteria per stage had generally shorter OS and a higher recurrence of metastasis (HR = 2.966, 95% CI = 1.347-6.532, p = 0.007, and HR = 2.579, 95% CI = 1.033-6.434, p = 0.042, respectively). Furthermore, patients with right-sided tumors exhibited a shorter OS time (HR = 2.183, 95% CI = 1.113-4.282, p = 0.023), while those who received chemotherapy demonstrated a longer OS time (HR = 0.430, 95% CI = 0.206-0.901, p = 0.025). The recipience of chemotherapy, being in stage III, having a high stage risk, and having a right-sided tumor emerged as robust predictors of mortality. While only stage III and high-stage risk were identified as strong predictors of metastasis. Notably, other demographic variables (sex and age) did not exhibit any significant associations with the studied survival outcomes.

**Table 2:** Multivariable Cox regression model for variables association with OS. OS = overall survival; HR = hazard ratio; PH = proportional hazard

Variables	HR	95% CI for HR		P-value	P-value for the PH assumption test
		Lower	Upper		
Chemotherapy	0.43	0.206	0.901	0.025	
Age	1.003	0.977	1.029	0.84	0.13
Stage					
III	2.968	1.46	6.036	0.003	0.28
II	(REF)	(REF)	(REF)		
Risk of stage					
High risk	2.966	1.347	6.532	0.007	0.33
Low risk	(REF)	(REF)	(REF)		
Gender					
Female	0.599	0.29	1.24	0.168	0.65
Male	(REF)	(REF)	(REF)		
Site					
Right	2.183	1.113	4.282	0.023	0.35
Left	(REF)	(REF)	(REF)		

**Table 3:** Multivariable Cox regression model for variables association with disease-free Survival HR = hazard ratio; PH = proportional hazard

Variable	HR	95% CI for HR		P-value	P-value for the PH assumption test
		Lower	Upper		
Stage					
III	3.837	1.538	9.575	0.004	0.63
II	(REF)	(REF)	(REF)		
Risk of the stage					
High risk	2.579	1.033	6.434	0.042	0.16
Low risk	(REF)	(REF)	(REF)		
Gender					
Female	0.659	0.286	1.519	0.327	0.72
Male	(REF)	(REF)	(REF)		

## Discussion

In this retrospective observational study conducted at the Royal Medical Services Military Cancer Center in Jordan, we focused on assessing the demographic and clinicopathological characteristics of CRC patients and their impact on OS and DFS. The study’s primary outcomes involved understanding the factors influencing patient survival rates and identifying significant predictors associated with CRC progression.

OS rates after one year of follow-up reached 85% following surgical resection, with a significant association of better prognosis in those who underwent adjuvant chemotherapy. These findings were aligned with the literature, showing a survival benefit for the addition of chemotherapy in resected stage III CRC patients of 22-32% and a 30% reduction in recurrence risk, while it is unclear whether all stage II colonic adenocarcinoma patients benefit from adjuvant chemotherapy, but those with high-risk disease may benefit [11,12]. Furthermore, in a pooled analysis of 3,302 stage II-III CRC patients comparing adjuvant chemotherapy of fluorouracil to surgery alone, Gill et al. using multivariate analysis that adjuvant therapy had a better treatment outcome across all CRC stages, in addition to significantly higher benefit in stage III CRC patients [13]. Indeed, adjuvant treatment only provided a substantial advantage to a maximum of 30% of individuals. Among them, 50% had already achieved recovery through surgery, while 20% encountered disease relapse despite undergoing adjuvant treatment [14].

Univariate Cox models revealed significant associations between various clinicopathological characteristics and survival outcomes. The disease stage was associated with patients diagnosed with stage III CRC, and those classified as high risk within each stage demonstrated poorer OS and increased recurrence of metastasis. These findings are concordant with the results of Dienstmann et al. who investigated stage II-III CRC patients and showed a significant decrease in survival rates in stage III CRC patients, which was accompanied by lymph node metastasis [15]. Furthermore, Ying et al., in their study of 1,181 stage I-III CRC patients, showed that persistent high inflammation levels reduce the effectiveness of chemotherapy and play a role in the unfavorable outlook for individuals with stage III surgical CRC [16].

Tumor-sidedness has been shown to be a significant predictor of patient survival. Right-sided tumors were associated with poorer outcomes compared to left-sided tumors. A recent randomized controlled trial by The Japan Clinical Oncology Group (JCOG) Colorectal Cancer Study Group (JCOG2003A) showed that primary tumor sidedness was significantly associated with patient survival after tumor recurrence (HR = 0.77), but no significant association was found in relapse-free survival [17]. However, significant focus has been directed toward the molecular distinctions between right-sided colon cancer and left-sided colon cancer. In a recent meta-analysis by Gholamalizadeh et al. on the prognostic significance of primary tumor location in 1,494,445 CRC patients from 60 cohorts, right-sided colon cancer was significantly associated with decreased OS in advanced stages only (stage III-IV), with no significant difference in early stages I-II [18]. Those differences between proximal (right) and distal (left) carcinomas were associated with distinct molecular features and prognostic effects [19]. For instance, right-sided colon cancer was associated with high microsatellite instability (MSI-high) and mutations in tumorigenic pathways such as *BRAF *mutations. Whereas left-sided colon cancer was more likely associated with *EGFR *mutations and HER2 amplification [20]. Furthermore, the *KRAS *mutation stands out as the most extensively recognized oncogene, displaying the highest frequency of mutations across diverse cancer types. It is recognized as the predominant gene responsible for driving oncogenic processes in human cancers, notably prevalent in CRC, non-small cell lung cancer (NSCLC), and pancreatic cancer [21]. In Jordanian samples of CRC patients, wild-type *KRAS *was seen in 57% of patients, while 43% were *KRAS *mutant and showed significantly worse survival outcomes (HR = 2.045 [1.3-3.2]) compared to wild-type *KRAS *[22].

Our findings also suggest that stage III CRC patients were associated with worse DFS rates compared to stage II CRC patients. Interestingly, about 20% of CRC cases were at stage IV with known distant metastases. This suggests the need for precise risk assessment to guide pre and postoperative decisions, as patient outcomes after distant metastasis surgery vary greatly [23,24].

Despite the comprehensive insights gained from this study, certain limitations should be acknowledged. The retrospective nature of the study could lead to selection bias and incomplete data. Moreover, other potential prognostic factors, such as molecular characteristics of tumors or specific treatment regimens, were not explored. In addition, due to the limited resources available, MSI status was not evaluated in or study.

## Conclusions

This study confirms the importance of factors such as cancer stage, risk levels, chemotherapy, and tumor location in shaping the prognosis of CRC patients. These findings hold significant implications for clinical practice, emphasizing the necessity for tailored interventions and strategies to enhance survival rates and quality of life for CRC patients, not only in Jordan but also in similar healthcare contexts. Further research, considering additional prognostic factors and potentially employing prospective designs, could provide a more comprehensive understanding of CRC outcomes and guide more effective treatment approaches.
